# A biofluid-repellent nanograss coating enhances flow of protein solutions and preserves transparency of glass capillaries upon exposure to blood

**DOI:** 10.1039/d5na00433k

**Published:** 2025-11-07

**Authors:** Mubashir Hussain, Mohammad Awashra, Christoffer Kauppinen, Seyed Mehran Mirmohammadi, Nicholas Addy-Tayie, Rosa Peltola, Juho Leskinen, Sami Puustinen, Heikki A. Nurmi, Robin H. A. Ras, Sami Franssila, Ville Jokinen

**Affiliations:** a Department of Chemistry and Materials Science, School of Chemical Engineering, Aalto University Tietotie 3 Espoo 02150 Finland mubashir.hussain@aalto.fi ville.p.jokinen@aalto.fi; b Quantum Photonics Research Team, VTT Technical Research Centre of Finland Ltd Tietotie 3 Espoo FI-02044 VTT Finland; c University of Eastern Finland, Faculty of Science, Forestry and Technology Joensuu Finland; d Kuopio University Hospital, Microsurgery Center of Eastern Finland Kuopio Finland; e Department of Applied Physics, School of Science, Aalto University Espoo Finland; f Centre of Excellence in Life-Inspired Hybrid Materials (LIBER), Aalto University Espoo Finland

## Abstract

A transparent and superhydrophobic (SHB) nanograss coating makes glass capillaries repellent toward protein solutions and blood. The coating is fabricated using atomic layer deposited alumina which is converted into grass-like alumina (GLA) by hot water treatment (HWT). The resulting tubes are transparent and highly repellent towards water and protein solutions (sliding angle <6° for a concentrated albumin solution). The biofluid repellence of the coating reduces hydraulic resistances for protein-rich biofluids, including fetal bovine serum, by 0–50% with the strongest effect for the smallest tubes and lowest Reynolds numbers. This work addresses a knowledge gap where previous studies have mostly focused on pure water for the drag reduction effect. The tubes also show a promising ability to stay clean and transparent on short term exposure to blood, unlike unmodified glass or polymer controls.

## Abstract 2

Superhydrophobic (SHB) glass capillaries are fabricated by depositing thin alumina and its conversion into grass-like alumina. The capillaries repel water and biofluids including blood which indicates their applications in blood contacting devices.

## Graphical abstract

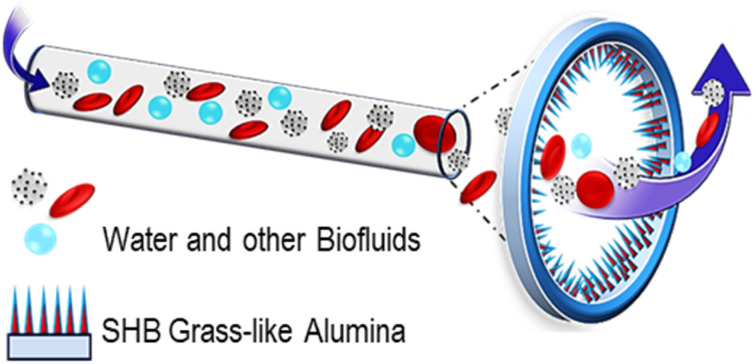

## 1. Introduction

Water repellent properties of the lotus leaf (lotus effect)^1^ have inspired the fabrication of many artificial liquid repelling surfaces.^2^ These surfaces are attractive for their technological applications such as self-cleaning,^3^ anti-icing,^4^ antifouling,^5^ anti-fogging^6^ anti-reflection,^7^ manipulated droplet transport,^8,9^ blood anti-adhesion^10^ and many others. SHB surfaces trap pockets of air (*i.e.*, plastron) between the applied liquid and the micro/nanostructures forming what is called a Cassie–Baxter wetting state. The air plastron lowers the surface energy by minimizing the liquid–solid interface where the liquid will be in partial contact with the surface roughness peaks and air.^11^ Naturally occurring SHB surfaces (*e.g.*, plant leaves) have been reported to exhibit a water contact angle of >150° and contact angle hysteresis of less than 10°. The superhydrophobicity of these plants is caused by surface features at a scale of 10 μm which are decorated with 100 nm asperities as described by Barthlott and Neinhuis.^12,13^

A number of materials and fabrication techniques have been reported to prepare SHB surfaces.^14^ Planar SHB surfaces have also been successfully fabricated by using grass-like alumina (GLA) coated with a plasma-deposited fluoropolymer^15^ or silane treated GLA.^16^ GLA is a nanorough coating, produced by hot water treatment (HWT) of atomic layer deposition (ALD) deposited alumina thin films, originally used in optical applications as a reflection suppressing coating.^17^ GLA exhibits a grass-like morphology that increases the surface area and forms a naturally gradient-refractive index profile, as the structure is denser at the nanograss “roots” than at the “tops”. This gradient suppresses optical reflections on glass, and the coating can be deposited conformally at low temperature.^17^ Due to these desirable properties, GLA has been used in numerous applications such as a SHB antireflection coating,^15,16^ a high specific surface-area capacitance enhancing supercapacitor nanoelectrode,^18^ an omnidirectional antireflection coating on packaged black-silicon photodiodes,^19^ and a performance enhancing layer in transparent Al-doped ZnO thermoelectric films.^20^ However, GLA SHB coatings have not been used previously to make complex shaped SHB components like SHB tubes or capillaries. While ALD–HWT GLA coatings have been demonstrated on planar substrates, their conformal extension into transparent tubular geometries and validation in protein-rich and blood environments, as shown here, has not been previously reported.

Fabrication of tubes and capillaries with an SHB inner surface has been significantly more challenging than flat surfaces. Tubes and capillaries with tailored wetting properties could be highly desirable for medical devices,^21^ gas separation,^22^ water filtration,^23^ and microfluidics.^24^ Biofluid repellency can help medical devices in reducing biofouling which eliminates bacterial contamination and sustains optical transmittance in transparent devices. Wang *et al.* fabricated flexible PDMS SHB tubes by using a micron size structured tube template inserted in a larger diameter tube.^25^ The space between the two tubes was filled with PDMS. After curing, the template and outer tubes were removed and a SHB PDMS tube was obtained. Hoshian *et al.* fabricated PDMS/titania composite tubes that repel water and blood using nanorough aluminum tube templates.^26^ SHB tubes have also been obtained by applying a SHB coating on the inner surface of the tubes. Sun *et al.* recently reported polyvinyl chloride SHB tubes by applying a mixture of hydrophobic materials into the tube.^27^

SHB tubes show little friction to movement of droplets, and they can also reduce the resistance of liquids flowing through them. When a liquid flows over an SHB surface, the presence of plastron replaces the solid–liquid interaction with liquid–gas interaction which results in less viscous losses at the surface. Consequently, the velocity of the fluid does not reach zero at the walls, leading to the emergence of slip. The slip length depends on the hydrophobicity of the surface; specifically, the more (super)hydrophobic the surface, the greater the slip length, and *vice versa*.^28,29^ A number of studies focusing on application of planar SHB surfaces for drag reduction have been performed and reviewed recently by Liravi *et al.*^30^ The drag reduction in the tubes allows liquid to flow more easily at a given pressure, effectively lowering their hydraulic resistance. Kim *et al.* fabricated structurally modified PDMS SHB tubes and compared the drag reduction behavior with smooth PDMS. Their results showed that structured SHB tubes could reduce the drag force at low Reynolds number more effectively.^31^ Geraldi *et al.* reported the fabrication of smooth and rough mesh pipes by a coating method. Rough mesh SHB pipes were capable of supporting plastron and subsequently resulted in higher drag reduction as compared to those with smooth surface pipes.^32^

Drag reduction studies performed to date have mainly used water and much less is known about the drag reduction of proteins and other biological fluids. Protein adsorption is commonly considered to be the first event following blood-material contact.^33^ When medical devices encounter the blood, the surface charge activates the protein factor XII present on the platelets. Thrombin starts to polymerize fibrinogen and results in clot formation within minutes.^34,35^ To prevent blood clotting, anticoagulants such as sodium citrate, citric acid, lactic acid and heparin have been reported, yet they could slowly leach and interfere with medical procedures and patient medication.^36^ An alternate solution to meet the challenge of blood coagulation is to utilize a surface which can minimize the contact with blood. SHB surfaces with different textures have shown the ability to repel proteins, platelets, and cells, thereby minimizing blood adhesion and preventing coagulation.^37,38^ Protein adsorption in biomedical devices is generally not desirable as it can lead to cell adhesion, blood coagulation, and device contamination. Therefore, it is highly desirable to explore the ability of transparent SHB tubular surfaces to enhance drag reduction of biofluids with subsequent application of such surfaces in medical and clinical devices.

Here, we present the fabrication of a SHB nanograss coating inside glass capillaries to address the lack of transparent, anti-fouling tubing compatible with complex biological fluids. The coating is formed through atomic layer deposition, hot water treatment, and silanization, resulting in a conformal, low-adhesion coating throughout the tube. Unlike previous studies, which focused on pure water, our capillaries exhibit substantial hydraulic resistance reduction for protein-rich solutions. The tubes can also stay clean and transparent on short-term exposure to blood, unlike glass or polymer controls.

## 2. Experimental section

### 2.1. Fabrication of SHB tubes and monitor Si chips

Glass capillary tubes (Duran borosilicate glass) and Si wafer chips (1 × 1 cm) were cleaned by ultrasonication in isopropanol (IPA) and water and subsequently deposited with amorphous ALD alumina using a BENEQ TFS500S atomic layer deposition (ALD) tool. The reactor temperature and pressure were set at 120 °C and 5 mbar, respectively. The pulse time for trimethylaluminum (TMA) and water was 200 ms. In addition, nitrogen gas flow was maintained at 150 sccm into the reactor throughout the deposition process. The number of cycles was set at 340. Glass tubes and Si wafer pieces (chips) were coated with ALD Al_2_O_3_ in the same batch to ensure identical deposition conditions. The thickness of the coating on both monitor Si wafer pieces was measured before and after HWT by ellipsometry (Plasmos He–Ne Ellipsometer).

HWT procedure for GLA was carried out in an IKA HBR 4 digital heat bath. HWT process is as follows: the temperature of the heat bath was first stabilized to 90 °C and ALD Al_2_O_3_ coated samples (glass capillary tubes and monitor Si chips) were immersed into water for 30 minutes. This process facilitated the transformation of the deposited Al_2_O_3_ layer into GLA.

**Fig. 1:**
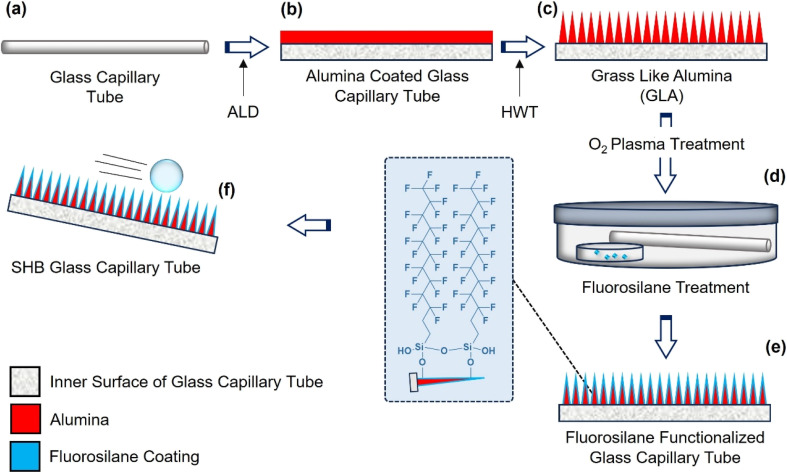
Schematic illustration of the fabrication process of SHB glass capillary tubes. (a) Clean glass capillary tube, (b) coating of alumina on the walls of the tube using ALD, and (c) conversion of the ALD alumina layer into GLA by simple HWT. O_2_ plasma treatment activates the GLA surface and (d and e) gas phase silanization covers the GLA with the hydrophobic fluorosilane layer. (f) SHB tube which repels water and other biofluids.

GLA was then functionalized with the self-assembled monolayer of 1*H*, 1*H*, 2*H*, 2*H*, trichloroperfluorododecylsilane (TPFS). TPFS was purchased from Good Laboratory Practice Bioscience (GLPBIO). The surface functionalization begins with activation of the surface of the samples. Oxygen plasma treatment of the samples was carried out using a Tepla 400 plasma tool. The treatment recipe was plasma power 40 W at an oxygen flow of 500 mL min^−1^ for 2 minutes. Gas phase silanization was carried out immediately after O_2_ plasma treatment. For silanization, O_2_ plasma treated samples were placed in a glass container housing a small Petri dish containing ≈35 μg TPFS (Fig. 1). The container was then heated on a hot plate at 100 °C for 1 hour. SHB glass capillary tubes and SHB Si chips were obtained.

The surface morphology of the fabricated SHB glass tubes and monitor Si chips was studied by using a scanning electron microscope (EBL Zeiss Supra 40). 10-cm long glass tubes were divided into 5 equal sized pieces with 2 cm each. SEM studies were carried out for two end pieces and a central piece. Glass pieces of each section were cleaned and sputtered with an 11-nm thick layer of gold prior to SEM studies.

### 2.2. Wettability characterization

Static and dynamic contact angle measurements were carried out using a contact angle goniometer (Theta, Biolin Scientific) to evaluate the wetting behavior of fabricated SHB surfaces. A 2 μL deionized water droplet was dropped on the surface with the help of a goniometer needle and the contact angle was recorded. Advancing and receding contact angles were measured from 2 μL to 5 μL and 5 μL to 2 μL volume of water at a flow rate of 0.1 μL s^—1^, respectively. Measurements were taken at three random spots of the fabricated surfaces and an average was reported.

The angle at which a liquid droplet starts to slide across the surface is known as the sliding angle. The sliding angle for water droplets (10 μL) and BSA solution was measured by using an in-house built sliding angle goniometer setup. SHB capillary tubes or Si monitor chips were placed on the top of the leveled goniometer. The sliding angle was quantified by gently placing the water/biofluid droplet of desired volume on the Si monitor chip or inside the SHB capillary tube. The sample was then gently tilted until the droplet started to slide. Three readings were taken, and an average was reported.

### 2.3. Scanning droplet tribometer (SDT) measurements

The scanning droplet tribometer is based on the oscillating droplet tribometer.^39,40^ Both of these instruments scan the sample surface with a ferrofluid droplet, which is moved with a magnetic potential well created with two permanent magnets and whose movement is captured with a camera. The SDT moves the magnets in a slow scanning motion, which allows the ferrofluid droplet to follow the magnetic potential with a small displacement. This ferrofluid droplet displacement (Δ*x*) from the potential well minimum (magnet axis) causes a magnetic spring force (*F*_m_ = −*k*Δ*x*). This spring force is equal to the friction force that the droplet experiences when the droplet is moving with a constant velocity (including staying still). The spring coefficient (*k*) was calibrated by oscillating the ferrofluid droplet in the magnetic potential and calculating the spring coefficient of the potential well based on the oscillation frequency. The displacement (Δ*x*) was calculated by assuming that the magnet axis is the median location of the measured droplet location over the back-and-forth measurements. The used ferrofluid was created in-house based on the coprecipitation method and stabilized with citric acid near pH 7.^41,42^ The ferrofluid was then left to evaporate until reaching 6.6 vol% of magnetite nanoparticles and then diluted to 1.0 vol% for the experiment.

### 2.4. Biofluid preparation

All biofluids were freshly prepared. Three biofluids were prepared for this study: 2 mg mL^−1^ and 50 mg mL^−1^ of bovine serum albumin (BSA, Fisher Bio Reagents™) protein dissolved in deionized (DI) water, in addition to the RPMI 1640 cell medium (Gibco™) containing 10% fetal bovine serum (FBS) (Gibco™). All biofluids had 1% penicillin–streptomycin (10 000 U mL^−1^, Gibco™). BSA powder was dissolved in DI water by stirring for 10 min at room temperature. The full cell media solution was prepared by pipetting FBS into the RPMI 1640 media in a biosafety laminar hood.

### 2.5. Hydraulic resistance measurement

The experimental setup for the flow rate measurement is shown in Fig. S1. SHB and control tubes were inserted in the wall of a plastic laboratory beaker. Holes matching the outer diameter of the four tube sizes were drilled at 10 mm above the bottom of the beaker. For real-time mass measurement of the flowing fluid, an electronic balance with a receiving container was set next to the connected tube outlet. The mass and time to collect the fluid were recorded using a camera. All tubes were planarized at around 0-degree tilt and fixed at that position before starting the experiment. The fluid collected in the receiving container was poured back into the reservoir to keep the initial conditions identical. The experiments were a combination of four fluids; four tube sizes; and two tube types (control and SHB tubes). Three repetitions were performed for each experiment, and the mean value was reported. The reported datapoints are at matched pressures within the experimental accuracy. Due to the possibility of differing capillary pressure in SHB *vs.* control tubes, we have chosen to use data only down to 50 Pa.

### 2.6. Protein adsorption experiments

Albumin–fluorescein isothiocyanate conjugate (Sigma Aldrich, FITC-labeled BSA) was used to investigate protein adsorption on tubes. First, a calibration curve (Fig. S2) was established to convert fluorescence intensity into the corresponding amount of adsorbed protein. A series of FITC-BSA solutions were prepared with RPMI 1640 as the solvent. Next, 5 μL of each concentration was applied onto glass slides and allowed to dry. Fluorescent images were captured using a Nikon Eclipse fluorescence microscope at 10× magnification with a FITC filter. Subsequently, FITC-BSA was mixed with freshly prepared 10 mg mL^−1^ of non-labeled BSA dissolved in RPMI 1640 (pH 7.4). This protein solution was introduced into control and SHB tubes (6.0 mm in size) using three different application methods: droplet mode – a 10 μL droplet was placed on the inner wall of the tube; dynamic mode – the protein solution was continuously flushed through the tube in a setup similar to Fig. S1; static mode – the tubes were filled with the protein solution and sealed. After a defined contact time in each mode, the solution was removed, and the tubes were rinsed three times with deionized water. Finally, the tubes were dried and analyzed using fluorescence microscopy.

### 2.7. Fluorescence and blood contamination experiments

Whole venous blood was collected by a medical doctor (Sami Puustinen) at the Kuopio University Hospital. The present study was carried out with the approval of the Wellbeing Services County of North Savo Ethics Committee (#1299/2022) and informed consent was asked from the participants. The study followed the institutional guidelines of Kuopio University Hospital and the Declaration of Helsinki for medical research involving human subjects. The collected blood was aspirated through the control tube (uncoated), ultrasonic aspirator tube (Stryker Sonopet), conventional surgical suction tube and SHB tube by using a suitable surgical suction device (Medela Dominant). Blood contamination of tubes was evaluated by visual inspection. The tubes were then flushed with tap water and visually inspected.

Fluorescent tissue phantoms were prepared to simulate cancerous tissue.^43^ Asymmetrical (1 × 0.5 × 0.5 cm) tissue phantoms immersed in venous blood were aspirated using a surgical suction device. After establishing the detection signal baseline without a tube, the fluorescence intensity of the samples was measured through the different tubes using an aspirate tissue monitoring device.

## 3. Results and discussion

### 3.1. Fabrication and characterization of SHB glass capillary tubes

The fabrication process of SHB tubes utilizes GLA, a conformal nanorough coating with a large surface area and distinct grass-like morphology, treated with gas-phase silane. The fabrication process is schematically illustrated in Fig. 1. The process starts with atomic layer deposition of ALD alumina^44^ on glass tubes with different internal diameters (8.0 mm, 6.0 mm, 4.0 mm and 1.6 mm) but the same length (10.0 cm). The key steps of the fabrication process to achieve uniform superhydrophobicity inside the tubes are the ALD thin film deposition, HWT to convert the ALD Al_2_O_3_ film into GLA (Fig. 1a–c) and the hydrophobic gas phase silane coating (Fig. 1d and e). These steps create suitable roughness and surface chemistry needed for superhydrophobicity. This process is more conformal than the previously reported process of making SHB GLA using plasma deposited fluoropolymer on GLA^15^ and is similar to a recent report of silane treated GLA on planar glass.^16^

The tubes were loaded into the ALD reactor with a monitor planar silicon chip (cleaved from a polished, monocrystalline Si wafer) at both ends (see Fig. S3 in the SI). The purpose of the monitor Si chips was to be a conformality test of the ALD process. If the monitor chips at the tube entry and exit have different thicknesses then the ALD pulse lengths would be insufficient, and the process was in non-self-limited growth mode. If the monitor Si chips possess similar thicknesses, then the tube with a macroscopic (mm-scale) opening likely would have a conformal ALD alumina coating that could be verified later using scanning electron microscopy (SEM). Although ALD is often considered time-consuming, industrial-scale reactors (*e.g.*, Beneq P1500) and commercial deployments (*e.g.*, Forge Nano cathode coating systems) show that large-scale ALD production is already feasible.^45,46^ ALD Al_2_O_3_ coated tubes and monitor Si chips were then immersed in hot water (90 °C) for 30 minutes. This HWT transforms the ALD Al_2_O_3_ layer into GLA as depicted in Fig. 1c.

The resultant average ALD alumina film thickness at the inlet Si chip after ALD was 26.2 ± 0.3 nm, and the average thickness at the outlet Si chip was 25.1 ± 0.6 nm. The deposited thicknesses on the monitor chips just at the tube entry and exit are extremely similar. This indicates good conformality and excellent uniformity of the ALD process. After HWT, He–Ne ellipsometry was used to study the GLA formation on the Si chips. From He–Ne ellipsometry both inlet and outlet Si chips had films with an effective refractive index of roughly *n* ∼ 1.2 and the effective thicknesses were 122.5 ± 0.5 nm and 119.0 ± 2.6 nm respectively. These results are consistent with the referenced literature as Kauppinen *et al.* measured similar effective film properties for the gradient refractive index GLA film. Kauppinen *et al.* however noted that the actual GLA thickness in their studies is around 200 nm measured *via* SEM, as simple single layer ellipsometric models only give an effective refractive index and thickness for a gradient refractive index film like GLA. Processing of the tubes was continued, as there was high confidence in successful ALD and GLA formation, and SEM was later used to verify the presence of a conformal GLA film on the tube inner walls.^17^

**Fig. 2:**
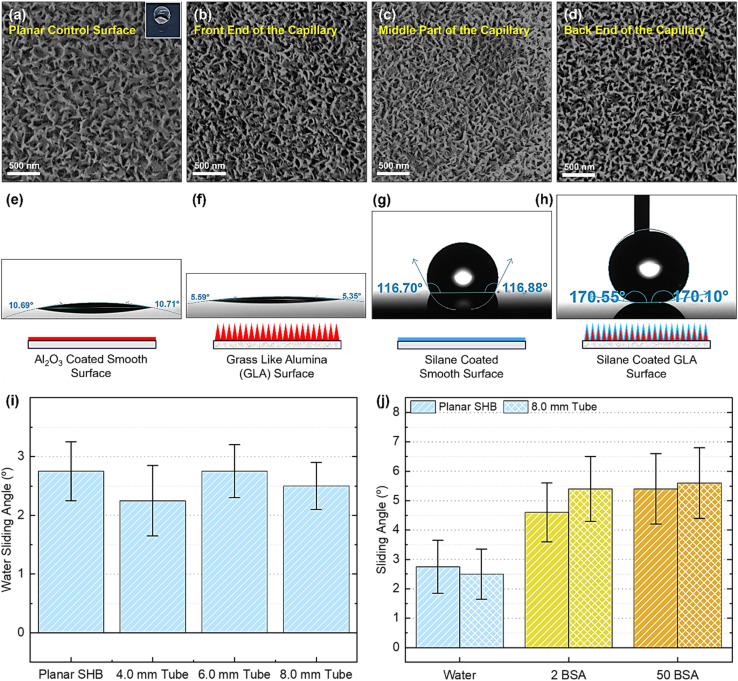
Surface characterization and wetting properties. (a) SEM image of the monitor Si chip placed at the end of the glass tube. A similar rough surface was observed for the hot water treated 8.0 mm glass tube as indicated in SEM images of the tube (b) front end, (c) middle part, and (d) back end. The inset in image (a) shows a water droplet on the SHB monitor Si chip. Water contact angle of (e) Al_2_O_3_ coated, (f) GLA surface, (g) silane coated smooth, and (h) silane coated GLA surface. (i) Sliding angle of water (10 μL droplet) for the SHB planar surface and three SHB tubes. (j) Sliding angles of water and 2 mg mL^−1^ (2BSA) and 50 mg mL^−1^ (50BSA) (indicated as 2BSA and 50BSA) protein solution (40 μL droplet) for the SHB planar surface and 8.0 mm SHB tube. Each data point in plot (i) and (j) represents the mean of three independent measurements (*n* = 3), and error bars represent the standard deviation.

The morphologies and wetting properties of the fabricated tubes are shown in Fig. 2. We studied the uniformity of the GLA inside the tubes. Fig. 2a shows the nanorough morphology of GLA on a planar Si chip. Fig. 2b–d show the GLA formation at the front end, middle and back end of the capillary tubes with 8.0 mm inner diameter. SEM investigations of the tubes with smaller inner diameters also showed rough surface morphology. Fig. S4 shows a cross-sectional SEM image of the GLA coating on the inner wall of the 6.0 mm glass tube. These results show successful and uniform GLA film formation on the inner walls of fabricated tubes and verify the indicative conformality result of the monitor Si chips.

The contact angles of the surfaces at various stages of the fabrication process (Al_2_O_3_ coated, Al_2_O_3_ nanograss, silane coated smooth, and silane coated nanorough surface Si chips) are shown in Fig. 2e–h. Contact angle measurements using the goniometer were not possible for glass tubes, therefore, the monitor Si chips were used. As monitor Si chips and glass tubes were coated under the same experimental conditions, and the monitor Si surfaces have a native oxide of silica that is chemically similar to glass, the ALD alumina growth is similar on glass and on the native oxide. We therefore assume that the contact angles of the inner walls of the tubes should be similar to the chips as the formed GLA on both chips and the tube inner walls should be nearly identical. The advancing contact angle for SHB Si chips was recorded to be 169° (±2°) while the receding contact angle was 168° (±1°) resulting in low contact angle hysteresis of 1° ± 2.2°. It indicates the successful fabrication of the SHB surface. The advancing contact angle obtained here is similar to a previous study where fluoropolymer coated GLA had an advancing contact angle of up to 173°.^15^ However, the contact angle hysteresis of the previous work was higher (12°) likely reflecting the different hydrophobic coatings.^15^ This shows the high quality of the obtained SHB surfaces in this work. ALD Al_2_O_3_ coated Si chips show a water contact angle of 10° ± 2° which decreased to 5° ± 1° upon transformation of smooth ALD Al_2_O_3_ into GLA (Fig. 2e and f). It can be attributed to the larger surface area provided by the GLA for better wetting leading to the Wenzel state. In contrast, GLA surface with silane coating showed a water contact angle of 169° ± 2° (Fig. 2h) which is the characteristic of SHB surfaces (Cassie state). It is worth mentioning that the Si chip simply coated with silane showed a water contact angle of 116° ± 2° (Fig. 2g). It implies that in addition to the silane coating, surface roughness plays an important role in the conversion of a hydrophobic surface into a SHB one. Entrapment of air (plastron) between GLA and water and reduction in surface energy further helps to bead up the water drop rendering the surface SHB.

Water and biofluid repellent properties of SHB capillary tubes were further characterized by sliding angle measurements. A very low sliding angle was measured for the SHB Si chips and SHB glass tubes (Fig. 2i and j). A 10 μL water droplet does not sit inside the tube and keeps rolling on slight tilting (SI Video SV1). A water droplet with 10 μL volume starts to slide in the tube when tilted at an angle of <3° (see Fig. 2i). The sliding angle for the smallest capillary (1.6 mm diameter) could not be measured due to its small diameter. The biofluid repellency of the tubes was also characterized by sliding angle experiments. Owing to the low surface tension, 10 μL bovine serum albumin (BSA) solution could not be transferred out of the pipette as a spherical drop. Therefore, higher volume of 40 μL BSA solution was dropped in the tube mouth. A sliding angle of <6° was measured for 2 mg mL^−1^ (2BSA) and 50 mg mL^−1^ (50BSA) solutions. The 50BSA solution slid off the SHB tube without leaving any stain (Fig. S5a–c). On the other hand, BSA solutions completely wet the control glass capillary tubes (Fig. S5d). Higher sliding angles for biofluids as compared to water could be attributed to lower surface tension of these solutions as measured previously.^47^ These results indicate that the fabricated SHB capillary tubes are not only water repellent, but they also show repellent properties towards biofluids.

**Fig. 3:**
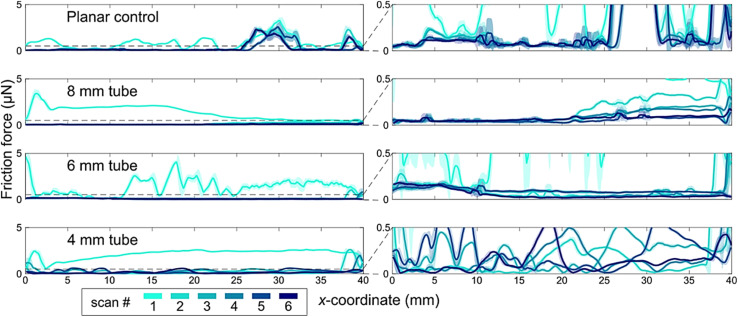
The scanning droplet tribometer friction force measurements on different samples with a 10 μL ferrofluid droplet with 1%-vol of iron nanoparticles. Each measurement consists of 3 back-and-forth line scans of 40 mm and the lines and shading are moving average and standard deviation of 50 points, respectively. The right column of subfigures is a rescaling of the data in the left column to 0.5 μN, which is shown with the dashed line. The tube samples of diameter 4, 6 and 8 mm were compared with a planar control sample.

Uniformity of the Al_2_O_3_ coating and subsequent superhydrophobicity was further evaluated by performing scanning droplet tribometer (SDT) measurements. This method scans the sample by moving a ferrofluid droplet with a magnetic potential well and determines the friction the droplet experiences based on the displacement of the droplet from the potential well minimum *i.e.*, magnet axis.^39,40^ The 50 point moving average and standard deviation of the friction between the droplet and the samples is shown in Fig. 3. The tube samples have similar friction values to the planar control samples in the low μN range. This indicates that the tubes have similar topography, chemistry, and wetting properties to a planar surface. All samples had a clear difference between the first and later scans with the first scan having roughly 10 times higher friction (around 2 μN) compared to the subsequent scans (around 0.2 μN). The reason for this is not clear, but the working hypothesis here is that the droplet cleans the surface with the first scan lowering the droplet friction for future scans. We also note that in most use cases the tube is either continuously in contact with water or biofluid, or else a priming step can be used, so the lower value is likely more relevant for practical use cases. All the samples are SHB from the friction force perspective, as the friction is similar to or lower than that of known SHB samples.^40,48,49^ Most samples are also homogeneous without spatial variability in the friction forces and very few pinning sites were observed inside the samples.

As shown in Fig. S6, SHB-coated tubes retained optical transparency at least as good as uncoated glass, consistent with our fluorescence transmission data (Fig. S7) and with prior reports using GLA coatings as an antireflection coating on glass with 99% average transmittance in a spectrum including the visible range.^17^

To evaluate short-term durability under sterilization-relevant conditions, the 6.0 mm tube was subjected to a standard biomedical protocol consisting of 15-min immersion in absolute ethanol followed by UV irradiation applied through the tube openings (as glass is not transparent to UV). The coating performance was unaffected, with the sliding angle of water remaining at about 3° before and after sterilization. These results indicate that the SHB nanograss coating can withstand basic sterilization treatment without loss of repellency, supporting its potential applicability in biomedical settings.

### 3.2. Hydraulic resistance of water and biofluids

The flow rates of water, protein solutions, and cell media solutions are compared to control (uncoated plain glass) and SHB tubes with four different internal diameters (1.6, 4.0, 6.0, and 8.0 mm) and the same length of 10.0 cm. The experimental setup is illustrated in Fig. S1 similar to the one reported in Kim *et al.*^31^ The % drag reduction was calculated by using the formula given in eqn (1).1
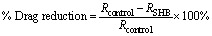
where *R*_control_ and *R*_SHB_ are the experimental hydraulic resistances of control and SHB glass tubes, respectively. Experimental hydraulic resistance (*R*_exp_) in the tubes was calculated using eqn (2).2
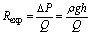
where, Δ*P* is the difference in pressure between the liquid air interface and center of the tube under investigation, *ρ* is the density of water, *g* is gravitational acceleration, *h* is the height of liquid between the tube center and fluid air interface, and *Q* is the volumetric flow rate in the tube.

**Fig. 4:**
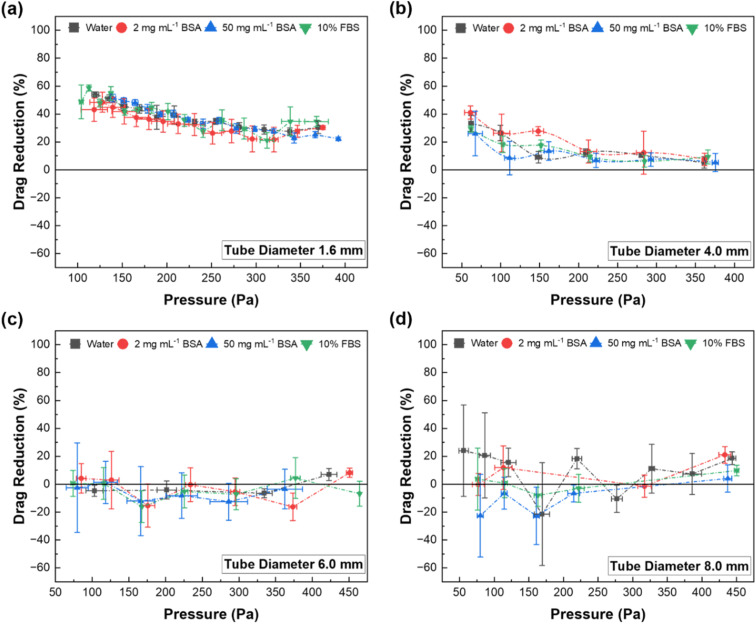
SHB glass tubes’ drag reduction as compared to the correspondent plain glass control tube. Each tube was exposed to different flow rates of water, 2 mg mL^−1^ BSA, 50 mg mL^−1^, and full cell media containing 10% fetal bovine serum (10% FBS). Glass tubes with (a) 1.6 mm, (b) 4.0 mm, (c) 6.0 mm, and (d) 8.0 mm internal diameters. Error bars represent the standard deviation of three independent measurements of each experiment (*n* = 3).

Fig. 4 shows the relationship between pressure and percentage drag reduction in SHB glass capillary tubes of varying diameters, relative to their correspondent plain glass control tube. The data reveal three key observations. The first observation is that the tube size has a clear effect on the drag reduction. The 6.0 mm and 8.0 mm tubes show no measurable drag reduction, whereas the two smaller tubes exhibit a clear drag reduction effect of at least 10% for all tested liquids at all tested pressures. The drag reduction effect in the SHB tubes is attributed to the trapped air plastron on the SHB walls which reduced the liquid–solid contact area and hence the frictional forces. The drag reduction effect is stronger for the 1.6 mm tube than for the 4 mm one, aligning with previous observations in the literature^50,51^ and theoretical models for the laminar flow of water.^52^ The noise levels for the two largest tubes are quite high due to the short time of each experiment in our set-up, but the data are clear that if there is any reduction for the large tubes, it is very minor.

The second observation is that the drag reduction effect seems to increase as the pressure, and consequently the Reynolds number, decreases. This observation is also supported by previous experimental results.^31,38,50,51,53–56^ For the 1.6 mm tube, the drag reduction starts at about 20% at 350 Pa (Re 400) and increases to 40% at 150 Pa (Re 200). A similar trend is observed for the 4.0 mm tube. The drag reduction is around 10% at 400 Pa (Re 1820) and increases to 20% at 100 Pa (Re 940). This value is consistent with Shirtcliffe *et al.*, who reported 30–50% drag reduction in 1.9 mm SHB-coated copper tubes with water. From the data we can also estimate the slip lengths using the slip-corrected Poiseuille flow model:^53^3
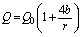
where *Q* is the flow rate in a superhydrophobic tube, *Q*_0_ the flow rate in the reference tube at the same pressure, *r* the radius of the tube and *b* the estimated slip length. For the 1.6 mm diameter tube, we get slip length estimates of 50 μm at the low end of 20% drag reduction and 130 μm at the high end of 40% drag reduction. For the 4 mm diameter tube, we get slip length estimates of 50 μm at the low end of 10% drag reduction, and 125 μm at the high end of 20% drag reduction. The slip lengths obtained are consistent between the tube sizes and are in the high end of the range of 1–100 μm that has been reported for the slip lengths of SHB surfaces.^52^ It is noteworthy that in both tubes, the flow remains within the laminar regime (Re <2000).

The third and most important observation is that the drag reduction effect seems to work equally well with all the tested biofluids as with pure water. There is no significant difference in our data between water and the biofluids in any of the four tube sizes, although we note that the exposure here was only short term. This seems to us to be a promising finding as it could significantly expand the use cases of SHB drag reduction in biomedical applications. There are scarce prior studies on SHB drag reduction of biofluids flowing in tubes. Li *et al.* demonstrated a blood drag reduction effect of up to 76% on a TiO_2_–SiO_2_-polydopamine composite surface, but this experiment was done with a planar surface using a rheometer.^57^

### 3.3. Protein adsorption

**Fig. 5:**
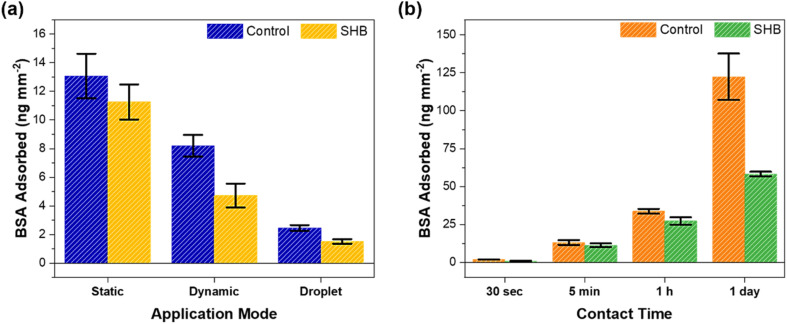
Bovine Serum Albumin (BSA) protein adsorption on uncoated and smooth control and SHB (coated nanograss) glass tubes. (a) Protein adsorption on control and SHB capillary tubes in static, dynamic and droplet application modes at a contact time of 5 min. (b) Protein adsorption on the control and SHB glass tubes at different contact times. Error bars represent the standard deviation of three measurements of each experiment.

The results shown in Fig. 5 illustrate the adsorption behavior of bovine serum albumin (BSA) protein on both plain control and SHB glass tubes under different conditions. Fig. 5a shows the comparison between control and SHB tubes using different modes of protein application onto the tube walls (static, dynamic, and droplet) for 5 min and suggests that surface superhydrophobicity significantly reduces BSA adsorption. Similar observations are found in the literature.^47,58–61^ SHB surfaces often prevent protein adsorption mainly due to their air plastron properties, forming a minimal contact area between the protein and the surface.^62^ Adsorption is lower in the dynamic mode (liquid is flowing through the tube) compared to the static mode (the tube is filled with the liquid and then capped), possibly due to increased shear forces in the flow motion and reduced protein–solid contact time. The droplet method had the lowest amount of adsorbed protein. In all cases, the adsorption on the SHB tube was slightly less than on the control tube.

Fig. 5b shows the time-dependent protein adsorption in the static mode. The data reveal that adsorption increases over time but at different rates for control and SHB tubes. Protein adsorption increases significantly for the control tube over time. Fluorescence microscopy images in Fig. S8 clearly show significantly lower fluorescence in SHB tubes after 24 hours of contact time as compared to the control tube. These results suggest that the air plastron lifetime of our SHB tubes is longer than 1 day making them potentially suitable for long-term exposure to at least protein solutions. Koc *et al.* reported that nanoscale roughness typically results in lower protein adsorption compared to smooth surfaces due to the limited surface contact area available for proteins to adsorb.^63^ They also demonstrated that exposure to flow shear significantly decreased protein adsorption compared to the no-flow (static) application mode on the surface.

### 3.4. Blood repellency and optical transparency

One possible application for transparent and SHB tubes is monitoring medical and surgical suction fluids visually and by optical detection methods. For example, in aspirate tissue monitoring during cancer surgery, optical detection through the suction tube provides near-real time feedback on tissue properties.^64^ The key requirement for this is optical transparency and non-fouling of the tube walls upon contact with the surgically removed material, including blood. We compared optical measurements through the 8.0 mm diameter SHB capillary tube, a plain 8.0 mm glass control tube, a Sonopet ultrasonic aspirator tube and a standard PVC polymer surgical suction tube. Fluorescent phantom tissue samples were immersed in human blood and aspirated through the tubes, while exciting the samples and detecting their fluorescent response. SI Fig. S7 shows the results of the fluorescent phantom tissue samples being aspired through the SHB glass capillaries and controls. The data have two components: the peaks are the fluorescent tissue samples passing through the sensor while the baseline is established on moments without a sample passing through the sensor. The data show that the SHB glass capillary is optically very close to the control glass capillary, which means that the optical properties are not degraded by the topography and hydrophobic coating procedures. The plastic control in contrast has a higher baseline. Overall, these results translate to reliable, high-specificity detection of the fluorescent signals.

**Fig. 6:**
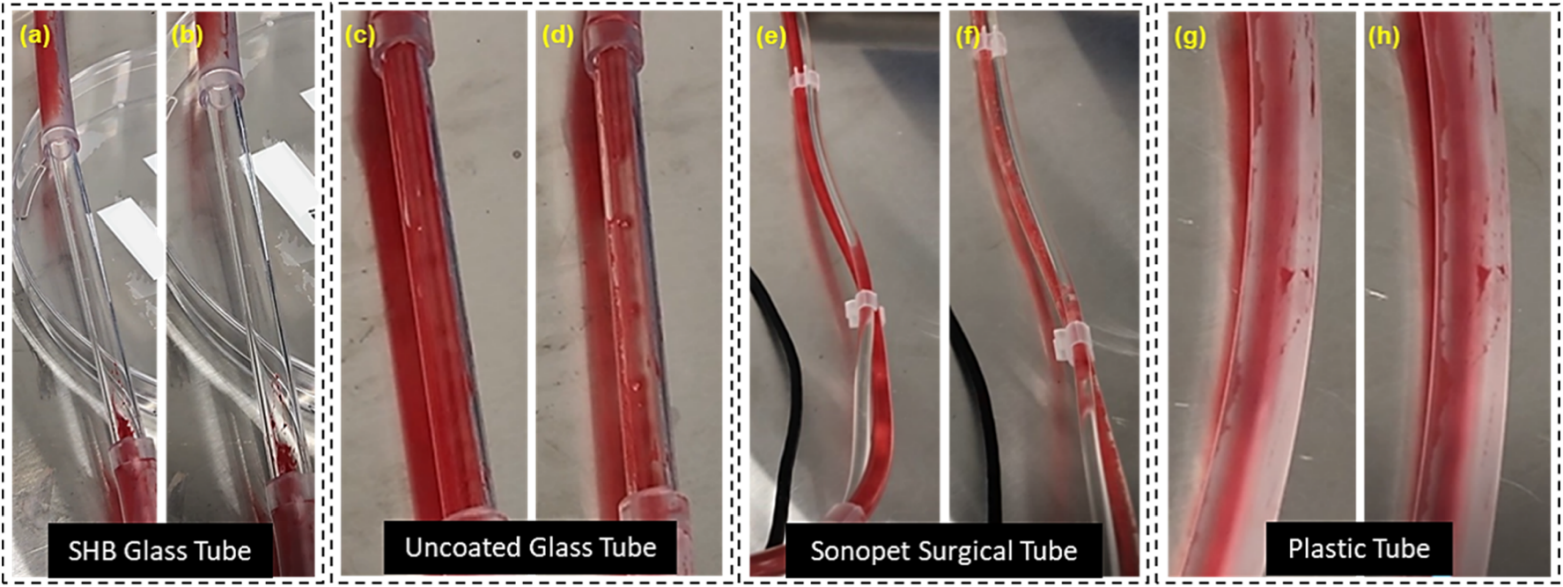
Blood contamination manifested by SHB glass, control glass, Sonopet surgical and control plastic tubes. Image (a), (c), (e) and (g) represent the respective tubes after blood flow had fully filled their entire cross section. (a) SHB tubes repel the blood and therefore no blood adherence was observed. Images (b, d, f and h): after flushing with air, blood adhesion was observed in control glass, Sonopet surgical and plastic tubes. No blood staining was observed in the case of SHB glass tubes.

Next, we visually compared the tubes upon exposure to blood (Fig. 6). Fig. 6a shows the SHB glass capillary tube after exposure to blood. The SHB glass capillary tube remains visually very clean while the Sonopet surgical tube (Fig. 6e) and plastic tubes (Fig. 6g) are completely contaminated. The tubes were then flushed with air. Fig. 6b shows the SHB glass capillary tube after flushing with air without much effect since the walls were already clear. Fig. 6d, f and h show the control glass capillary, Sonopet surgical and plastic tubes after flushing. It is evident that before flushing, the walls are coated with blood leading to a loss of transparency. Tubes were further flushed with water to see if transparency could be regained. After a separate flushing step, transparency could be regained although there was still some contaminating blood present. Fig. S9 shows that in control and other tubes, although some transparency is regained by flushing with water, they are not as transparent as the SHB capillary is. SHB tubes stay clean and do not require additional cleaning steps. Contamination by blood can be observed with the naked eye in all tubes except SHB capillaries. Thus, SHB glass capillary tubes outperform control glass, Sonopet and control plastic tubes. These results show that a transparent SHB capillary tube can remain optically clear, at least on short term exposure to blood.

## 4. Conclusion

We demonstrated a transparent, nanostructured SHB coating that renders glass capillary tubes repellent to water, proteins, and whole blood while preserving their optical transparency. The conformal grass-like alumina layer, fabricated *via* atomic layer deposition and hot water treatment, created a uniform repellent coating inside a 10-cm long capillary tube.

Unlike prior work, which focused on planar surfaces or pure water, we show that 10–50% drag reduction is possible also for biofluids, such as concentrated protein solutions. This result suggests that bio-repellent coatings could find use in biomedical devices where high flow rates delivered through as small tubes as possible are preferred. The coated tubes resisted biofouling during short term blood exposure and retained optical transparency, unlike glass and polymer controls. The SHB coating was sterilized using a standard biomedical sterilization protocol with ethanol and UV and its properties were not affected. These results together indicate potential use in blood contacting medical and diagnostic devices where transparency is required.

## Author contributions

M. H. and M. A. contributed equally to this work. M. H. fabricated and characterized all grass-like alumina-coated glass tubes, coated them with the hydrophobic fluorinated silane, and measured their sliding angles. M. A. performed the water and biofluid drag reduction experiment inside all glass tubes and conducted the protein adsorption study. M. H. and M. A. wrote the manuscript. C. K. developed the grass-like alumina (the transparent nanograss) deposition process on the tube inner surface. S. M. M., and N. A. T. conducted a preliminary study that developed a homogeneous coating of grass-like alumina into the glass tubes and measured their sliding angles. R. P. conducted a preliminary drag reduction assessment of the SHB glass tubes using water. J. L. and S.P. conducted optical transparency studies before and after blood exposure tests. H. A. N. and R. R. conducted scanning droplet tribometer (SDT) measurements and wrote the relevant part in the manuscript. S.F. supervised and gave valuable advice. V. J. designed the research plan, supervised the research, and edited the manuscript. All authors reviewed and approved the manuscript.

## Conflicts of interest

CK: Patent applications have been previously submitted related to this work including WO2019073111A1.

## Supplementary Material

NA-008-D5NA00433K-s001

NA-008-D5NA00433K-s002

## Data Availability

The data supporting this article have been included as a part of the supplementary information (SI). Supplementary information: experimental setup for measurement of flow rate of water and biofluids, calibration curve to convert FITC-labeled BSA fluorescence intensity to adsorbed amount of protein in ng mm^−2^, illustration of the arrangement of monitor Si chips and glass capillary tubes inside the ALD reactor for alumina coating, cross-sectional SEM image of the grass-like alumina coating on the inner wall of the glass tube, images showing wetting of the control and SHB tubes by bovine serum albumin solution, photographs representing optical transparency of control and SHB glass tubes, comparison of the optical measurements through the different tubes, fluorescence microscopy images of BSA adsorption on control and SHB tubes, photographs showing blood contamination of capillary tubes after flushing with water and a video showing real time observation of superhydrophobicity of the SHB glass capillary tube has been included in supplementary information section. See DOI: https://doi.org/10.1039/d5na00433k.

## References

[cit1] Koch K., Bhushan B., Barthlott W. (2008). Diversity of structure, morphology and wetting of plant surfaces. Soft Matter.

[cit2] Hu S., Cao X., Reddyhoff T., Puhan D., Vladescu S.-C., Wang J., Shi X., Peng Z., deMello A. J., Dini D. Liquid repellency enhancement through flexible microstructures. Sci. Adv..

[cit3] Lu Y., Sathasivam S., Song J., Crick C. R., Carmalt C. J., Parkin I. P. (2015). Robust self-cleaning surfaces that function when exposed to either air or oil. Science.

[cit4] Wang L., Gong Q., Zhan S., Jiang L., Zheng Y. (2016). Robust anti-icing performance of a flexible superhydrophobic surface. Adv. Mater..

[cit5] Zhang Y.-L., Xia H., Kim E., Sun H.-B. (2012). Recent developments in superhydrophobic surfaces with unique structural and functional properties. Soft Matter.

[cit6] Han Z., Feng X., Guo Z., Niu S., Ren L. (2018). Flourishing bioinspired antifogging materials with superwettability: progresses and challenges. Adv. Mater..

[cit7] Raut H. K., Dinachali S. S., Loke Y. C., Ganesan R., Ansah-Antwi K. K., Góra A., Khoo E. H., Ganesh V. A., Saifullah M. S. M., Ramakrishna S. (2015). Multiscale Ommatidial Arrays with Broadband and Omnidirectional Antireflection and Antifogging Properties by Sacrificial Layer Mediated Nanoimprinting. ACS Nano.

[cit8] Ben S., Zhou T., Ma H., Yao J., Ning Y., Tian D., Liu K., Jiang L. (2019). Multifunctional magnetocontrollable superwettable-microcilia surface for directional droplet manipulation. Adv. Sci..

[cit9] Jokinen V., Sainiemi L., Franssila S. (2008). Complex droplets on chemically modified silicon nanograss. Adv. Mater..

[cit10] Manabe K., Kyung K.-H., Shiratori S. (2015). Biocompatible slippery fluid-infused films composed of chitosan and alginate *via* layer-by-layer self-assembly and their antithrombogenicity. ACS Appl. Mater. Interfaces.

[cit11] Cassie A., Baxter S. (1944). Wettability of porous surfaces. Trans. Faraday Soc..

[cit12] Barthlott W., Neinhuis C. (1997). Purity of the sacred lotus, or escape from contamination in biological surfaces. Planta.

[cit13] Neinhuis C., Barthlott W. (1997). Characterization and distribution of water-repellent, self-cleaning plant surfaces. Ann. Bot..

[cit14] Parvate S., Dixit P., Chattopadhyay S. (2020). Superhydrophobic Surfaces: Insights from Theory and Experiment. J. Phys. Chem. B.

[cit15] Isakov K., Kauppinen C., Franssila S., Lipsanen H. (2020). Superhydrophobic Antireflection Coating on Glass Using Grass-like Alumina and Fluoropolymer. ACS Appl. Mater. Interfaces.

[cit16] An Z., Hao J., Sun S., Su J.-a., Yang C., Wang S., Cheng S., Dong B. (2024). Superhydrophobic Modification of Atomic Layer Deposition Antireflection Aluminum Oxide Film: A Simple Evaporative Coating Technique. Langmuir.

[cit17] Kauppinen C., Isakov K., Sopanen M. (2017). Grass-like alumina with low refractive index for scalable, broadband, omnidirectional antireflection coatings on glass using atomic layer deposition. ACS Appl. Mater. Interfaces.

[cit18] Isakov K., Sorsa O., Rauhala T., Saxelin S., Kallio T., Lipsanen H., Kauppinen C. (2022). Grass-like alumina nanoelectrodes for hierarchical porous silicon supercapacitors. Energy Adv..

[cit19] Kauppinen C., Pasanen T. P., Isakov K., Serué M., Heinonen J., Vähänissi V., Lipsanen H., Savin H. (2021). Grass-like alumina coated window harnesses the full omnidirectional potential of black silicon photodiodes. Appl. Opt..

[cit20] Koskinen T., Raju R., Tittonen I., Kauppinen C. (2023). Grass-like alumina enhances transmittance and electrical conductivity of atomic layer deposited Al-doped ZnO for thermoelectric and TCO applications. Appl. Phys. Lett..

[cit21] Luo J., Yu H., Lu B., Wang D., Deng X. (2022). Superhydrophobic Biological Fluid-Repellent Surfaces: Mechanisms and Applications. Small Methods.

[cit22] Yong J., Yang Q., Hou X., Chen F. (2022). Emerging Separation Applications of Surface Superwettability. Nanomaterials.

[cit23] Rasouli S., Rezaei N., Hamedi H., Zendehboudi S., Duan X. (2021). Superhydrophobic and superoleophilic membranes for oil-water separation application: A comprehensive review. Mater. Des..

[cit24] Si Y., Li C., Hu J., Zhang C., Dong Z. (2023). Bioinspired superwetting open microfluidics: from concepts, phenomena to applications. Adv. Funct. Mater..

[cit25] Wang J., Wu Y., Zhang D., Li L., Wang T., Duan S. (2020). Preparation of superhydrophobic flexible tubes with water and blood repellency based on template method. Colloids Surf., A.

[cit26] Hoshian S., Kankuri E., Ras R. H. A., Franssila S., Jokinen V. (2017). Water and Blood Repellent Flexible Tubes. Sci. Rep..

[cit27] Sun Z., Ding L., Tong W., Ma C., Yang D., Guan X., Xiao Y., Xu K., Li Q., Lv C. (2023). Superhydrophobic blood-repellent tubes for clinical cardiac surgery. Mater. Des..

[cit28] Dasaev M. , Trushin E., Kalakutskaya O. and Voloshenko A., On the reduction of hydraulic resistance based on the hydrophobization of functional surfaces, in Journal of Physics: Conference Series, IOP Publishing, 2021, vol. 2124, p. 012018

[cit29] Rothstein J. P. (2010). Slip on Superhydrophobic Surfaces. Annu. Rev. Fluid Mech..

[cit30] Liravi M., Pakzad H., Moosavi A., Nouri-Borujerdi A. (2020). A comprehensive review on recent advances in superhydrophobic surfaces and their applications for drag reduction. Prog. Org. Coat..

[cit31] Kim Y. W., Lee J. M., Lee I., Lee S. H., Ko J. S. (2013). Skin friction reduction in tubes with hydrophobically structured surfaces. Int. J. Precis. Eng. Manuf..

[cit32] Geraldi N. R., Dodd L. E., Xu B. B., Wells G. G., Wood D., Newton M. I., McHale G. (2017). Drag reduction properties of superhydrophobic mesh pipes. Surf. Topogr.:Metrol. Prop..

[cit33] Rabe M., Verdes D., Seeger S. (2011). Understanding protein adsorption phenomena at solid surfaces. Adv. Colloid Interface Sci..

[cit34] Furie B., Furie B. C. (1988). The molecular basis of blood coagulation. Cell.

[cit35] Stavrou E., Schmaier A. H. (2010). Factor XII: What does it contribute to our understanding of the physiology and pathophysiology of hemostasis & thrombosis. Thromb. Res..

[cit36] Scaravilli V., Di Girolamo L., Scotti E., Busana M., Biancolilli O., Leonardi P., Carlin A., Lonati C., Panigada M., Pesenti A. (2018). Effects of sodium citrate, citric acid and lactic acid on human blood coagulation. Perfusion.

[cit37] Jokinen V., Kankuri E., Hoshian S., Franssila S., Ras R. H. (2018). Superhydrophobic blood-repellent surfaces. Adv. Mater..

[cit38] Zhang Q., Dong J., Peng M., Yang Z., Wan Y., Yao F., Zhou J., Ouyang C., Deng X., Luo H. (2020). Laser-induced wettability gradient surface on NiTi alloy for improved hemocompatibility and flow resistance. Mater. Sci. Eng., C.

[cit39] Timonen J. V. I., Latikka M., Ikkala O., Ras R. H. A. (2013). Free-decay and resonant methods for investigating the fundamental limit of superhydrophobicity. Nat. Commun..

[cit40] Junaid M., Nurmi H. A., Latikka M., Vuckovac M., Ras R. H. A. (2022). Oscillating droplet tribometer for sensitive and reliable wetting characterization of superhydrophobic surfaces. Droplet.

[cit41] Massart R. (1981). Preparation of aqueous magnetic liquids in alkaline and acidic media. IEEE Trans. Magn..

[cit42] Vasilescu C., Latikka M., Knudsen K. D., Garamus V. M., Socoliuc V., Turcu R., Tombácz E., Susan-Resiga D., Ras R. H. A., Vékás L. (2018). High concentration aqueous magnetic fluids: structure, colloidal stability, magnetic and flow properties. Soft Matter.

[cit43] Lehtonen S. J. R., Vrzakova H., Paterno J. J., Puustinen S., Bednarik R., Hauta-Kasari M., Haneishi H., Immonen A., Jääskeläinen J. E., Kämäräinen O.-P. (2022). *et al.*, Detection improvement of gliomas in hyperspectral imaging of protoporphyrin IX fluorescence – *in vitro* comparison of visual identification and machine thresholds. Cancer Treat. Commun..

[cit44] Puurunen R. L. (2005). Surface chemistry of atomic layer deposition: A case study for the trimethylaluminum/water process. J. Appl. Phys..

[cit45] P1500 B , The world's largest ALD system for your biggest substrates

[cit46] Forgenano , ALD for Lithium-Ion Batteries

[cit47] Awashra M., Mirmohammadi S. M., Meng L., Franssila S., Jokinen V. (2025). Stable Air Plastron Prolongs Biofluid Repellency of Submerged Superhydrophobic Surfaces. Langmuir.

[cit48] Pilat D. W., Papadopoulos P., Schäffel D., Vollmer D., Berger R., Butt H. J. (2012). Dynamic Measurement of the Force Required to Move a Liquid Drop on a Solid Surface. Langmuir.

[cit49] Daniel D., Vuckovac M., Backholm M., Latikka M., Karyappa R., Koh X. Q., Timonen J. V. I., Tomczak N., Ras R. H. A. (2023). Probing surface wetting across multiple force, length and time scales. Commun. Phys..

[cit50] Hoshian S., Kankuri E., Ras R. H., Franssila S., Jokinen V. (2017). Water and blood repellent flexible tubes. Sci. Rep..

[cit51] Lv F., Zhang P. (2016). Drag reduction and heat transfer characteristics of water flow through the tubes with superhydrophobic surfaces. Energy Convers. Manage..

[cit52] Lee C., Choi C.-H., Kim C.-J. (2016). Superhydrophobic drag reduction in laminar flows: a critical review. Exp. Fluids.

[cit53] Shirtcliffe N. J., McHale G., Newton M. I., Zhang Y. (2009). Superhydrophobic copper tubes with possible flow enhancement and drag reduction. ACS Appl. Mater. Interfaces.

[cit54] Kavalenka M. N., Vüllers F., Lischker S., Zeiger C., Hopf A., Röhrig M., Rapp B. E., Worgull M., Hölscher H. (2015). Bioinspired air-retaining nanofur for drag reduction. ACS Appl. Mater. Interfaces.

[cit55] Zhang L., Wan X., Zhou X., Cao Y., Duan H., Yan J., Li H., Lv P. (2024). Pyramid-Shaped Superhydrophobic Surfaces for Underwater Drag Reduction. ACS Appl. Mater. Interfaces.

[cit56] Marlena J., Tan J. K. S., Lin Z., Li D. X., Zhao B., Leo H. L., Kim S., Yap C. H. (2021). Monolithic polymeric porous superhydrophobic material with pneumatic plastron stabilization for functionally durable drag reduction in blood-contacting biomedical applications. NPG Asia Mater..

[cit57] Li Q., Tang F., Wang C., Wang X. (2017). Novel mussel-inspired Ti-6Al-4V surfaces with biocompatibility, blood ultra-drag reduction and superior durability. Mater. Sci. Eng., C.

[cit58] Song W., Mano J. F. (2013). Interactions between cells or proteins and surfaces exhibiting extreme wettabilities. Soft Matter.

[cit59] Zhang J., Li G., Man J., Qu Y., Guo Z., Zhang S., Li D. (2021). Mechanism of anti-proteins adsorption behavior on superhydrophobic titanium surface. Surf. Coat. Technol..

[cit60] Wang Y., Zhang B., Dodiuk H., Kenig S., Barry C., Ratto J., Mead J., Jia Z., Turkoglu S., Zhang J. (2021). Effect of protein adsorption on air plastron behavior of a superhydrophobic surface. ACS Appl. Mater. Interfaces.

[cit61] Schulte C., Podestà A., Lenardi C., Tedeschi G., Milani P. (2017). Quantitative control of protein and cell interaction with nanostructured surfaces by cluster assembling. Acc. Chem. Res..

[cit62] Awashra M., Jokinen V. (2025). Superhydrophobic Cell-Repellent Microstructures: Plastron-Mediated Inhibition of A549 Epithelial Cell Adhesion. Small.

[cit63] Koc Y., de Mello A. J., McHale G., Newton M. I., Roach P., Shirtcliffe N. J. (2008). Nano-scale superhydrophobicity: suppression of protein adsorption and promotion of flow-induced detachment. Lab Chip.

[cit64] Elomaa A., Lehtonen S., von und zu Fraunberg M., Charbel F., Luoma J., Visuri M., Haapala I., Haapasalo J., Vik-Mo E., Puustinen S. P. (2024). A intraoperative aspirate tissue monitoring during 5-ala guided high-grade glioma surgery. Neuro-Oncol..

