# Supplementary material for: A biofluid-repellent nanograss coating enhances flow of protein solutions and preserves transparency of glass capillaries upon exposure to blood
Source: Nanoscale Adv. 2025 Nov 7;8(1):67–79. doi: 10.1039/d5na00433k (PMC12593835; doi:10.1039/d5na00433k)
Supplement: NA-008-D5NA00433K-s001 [file NA-008-D5NA00433K-s001.pdf]

Supplementary Information (SI) for Nanoscale Advances.
This journal is © The Royal Society of Chemistry 2025

Supplementary Information for

Biofluid-Repellent Nanograss Coating Enhances Flow of Protein Solutions and Preserves Transparency of Glass Capillaries upon Exposure to Blood

Mubashir Hussain,[a*#] Mohammad Awashra,[a#] Christoffer Kauppinen,[b] Seyed Mehran Mirmohammadi,[a] Nicholas Addy-Tayie,[a] Rosa Peltola,[a] Juho Leskinen,[c] Sami Puustinen,[d] Heikki A. Nurmi,[e] Robin Ras,[e] Sami Franssila,[a] Ville Jokinen [a,*]

[a] Department of Chemistry and Materials Science, School of Chemical Engineering, Aalto University, Tietotie 3, Espoo 02150, Finland.

[b] Quantum photonics research team, VTT Technical Research Centre of Finland Ltd, Tietotie 3, Espoo FI-02044 VTT, Finland.

[c] University of Eastern Finland, Faculty of Science, Forestry and Technology, Joensuu, Finland.

[d] Kuopio University Hospital, Microsurgery Center of Eastern Finland, Kuopio, Finland.

[e] Department of Applied Physics, School of Science, Aalto University, Espoo, Finland.

[#] These authors contributed equally to this work.

[*] Corresponding Author E-Mail(s): mubashir.hussain@aalto.fi, ville.p.jokinen@aalto.fi

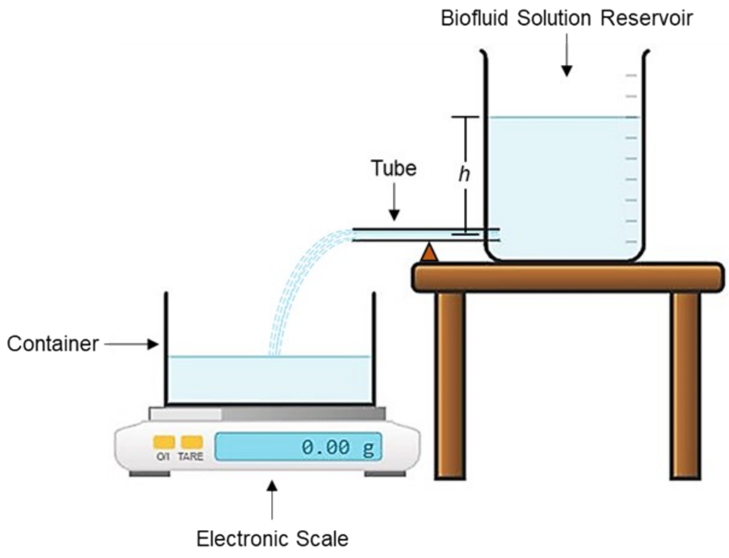

Figure S 1: Experimental setup for the measurement of flow rate of water and biofluids through control and SHB capillary tubes.

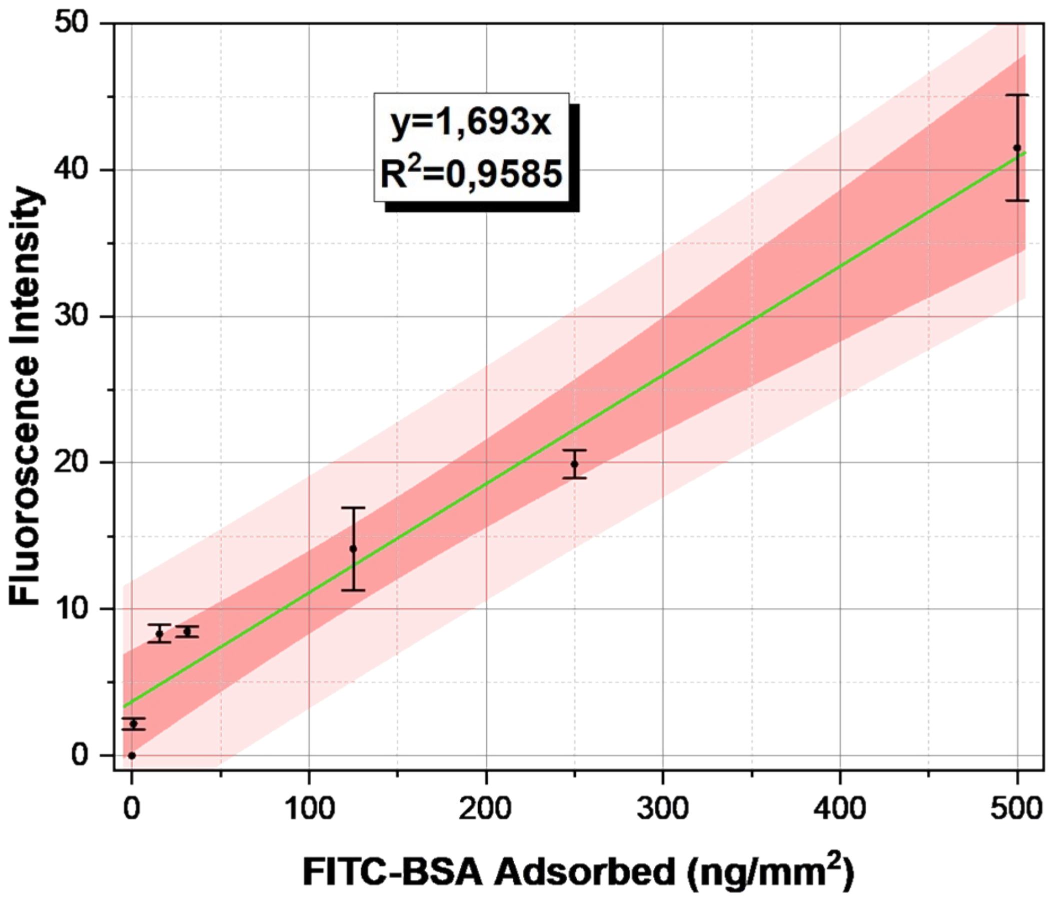

Figure S 2: A calibration curve to convert FITC-labeled BSA fluorescence intensity to adsorbed amount of protein in ng mm^{-2}.

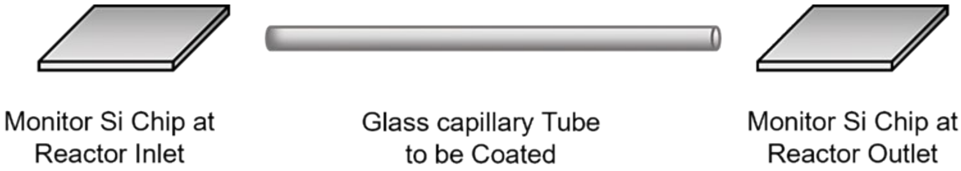

Figure S 3: Illustration of the arrangement of monitor Si chips and glass capillary tubes inside the ALD reactor for alumina coating.

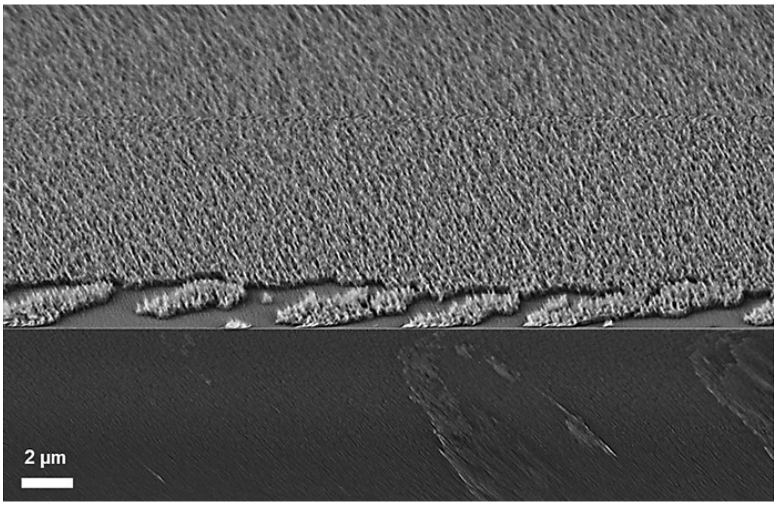

Figure S 4: Cross-sectional SEM image of the grass-like alumina coating on the inner wall of a 6.0 mm diameter glass tube (the loss of coating on the edge is due to breaking the tube in the middle). The coating shows uniform nanograss morphology across the cross-section of the middle of the tube, confirming conformal deposition inside the tubular geometry. Scale bar: 2 µm.

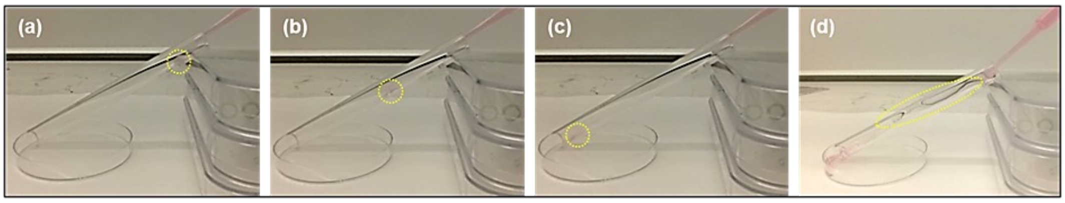

Figure S 5: Bovine Serum Albumin Solution inside Control and SHB Tubes. (a-c) BSA solution could not stick to the walls of SHB capillary tube and travelled in the form of droplet through the tube. In comparison, in image (d), control tube with flowing BSA solution is shown. BSA solution wets the walls of the control tube.

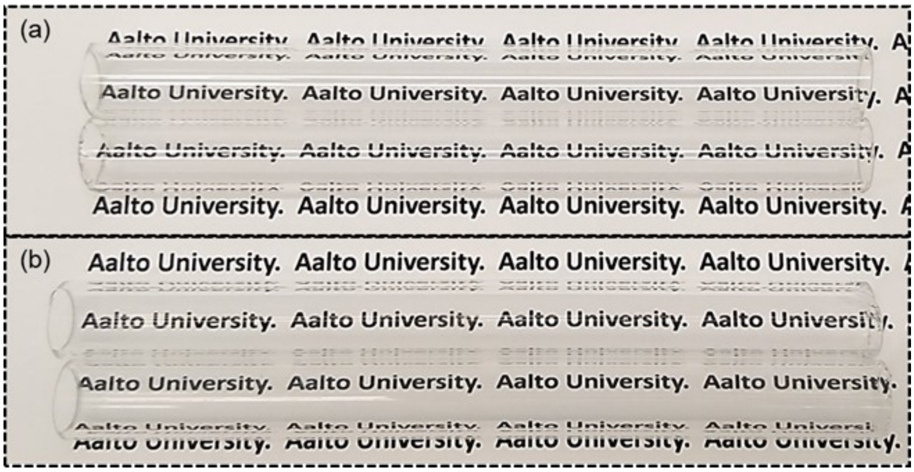

Figure S 6: Optical transparency of glass tubes. (a) Uncoated glass tube and (b) SHB nanograss-coated glass tube placed over printed text ("Aalto University"). Both tubes show transparency with the SHB coated tubes showing less reflection from ceiling lights, indicating that the SHB coating retains the antireflection property of GLA on glass and improves transparency and does not impair it.

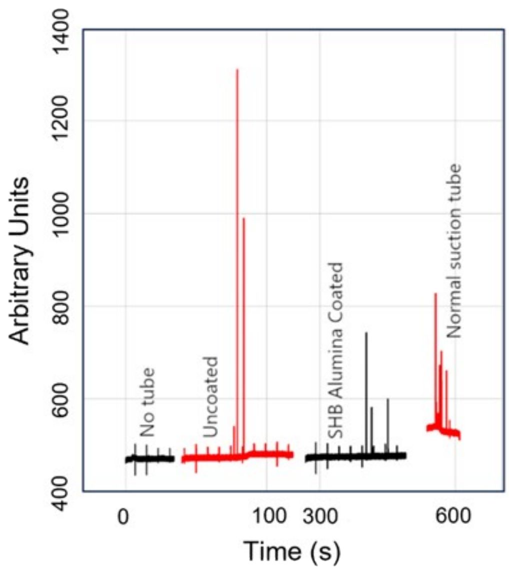

Figure S 7: Comparison of the optical measurements through the different tubes. The light intensity of the fluorescent samples flowing inside the tubes was measured by an aspirate tissue monitoring device (medical device). The "No tube" measurement is a reference baseline produced by the device. Uncoated = control glass capillary with no coating, SHB alumina coated = SHB alumina nanocoated glass capillary, and Normal suction tube = PVC bubble tubing conventionally used in surgical suction devices. The baselines of the signals were roughly 5-10% lower in the glass capillaries versus normal suction tubes. The coated glass capillaries and the uncoated control glass capillary had very similar signal baseline values (largest difference 478 vs. 470 ≈ 1.6%).

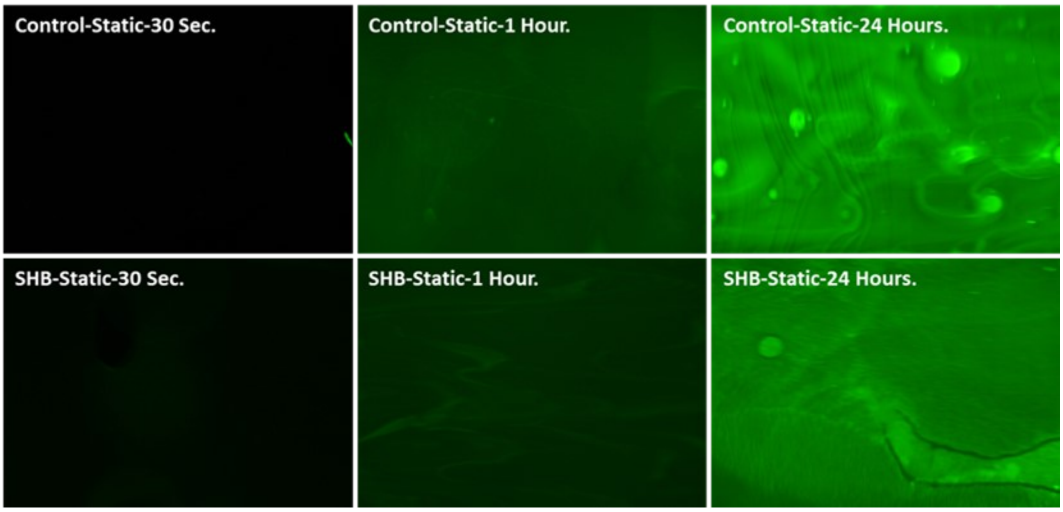

Figure S 8: Fluorescence microscopy images of BSA adsorption on control (uncoated) and SHB tubes. Images were taken at different time intervals i.e., 30 Seconds, 1 Hour and 24 Hours. Appreciable BSA nucleation can be seen in the case of control (uncoated) tubes as compared to coated SHB tubes after 24 hours.

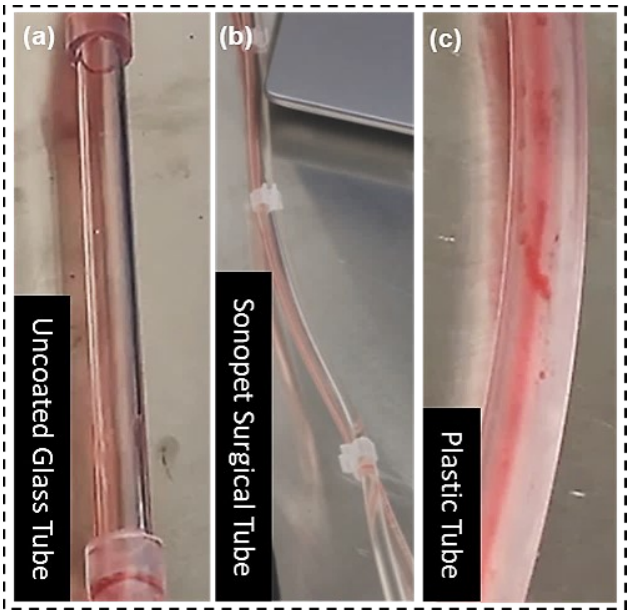

Figure S 9: Blood contamination studies of capillary tubes after flushing with water. In Control glass tube (a), Sonopet surgical tube (b) and plastic tube (c), some transparency is regained yet blood contamination is still observable with naked eyes.

Supporting Movie: SV1. Real time observation of superhydrophobicity of fabricated SHB glass capillary tube. A 10μL water drop does not spread on the surface of the tube. The rough grass like alumina (GLA) coated with silane traps the plastron below and beads up the water in the form of drop. The drop moves freely inside the tube on slight tilting without sticking which indicates the homogeneous and uniform superhydrophobicity inside the tube.
